# Supplementary material for: The Ultrasensitive Detection of Aflatoxin M1 Using Gold Nanoparticles Modified Electrode with Fe3+ as a Probe
Source: Foods. 2023 Jun 28;12(13):2521. doi: 10.3390/foods12132521 (PMC10340257; doi:10.3390/foods12132521)
Supplement: Supplementary file 1 [file foods-12-02521-s001.zip › foods-2454377-supplementary.pdf]

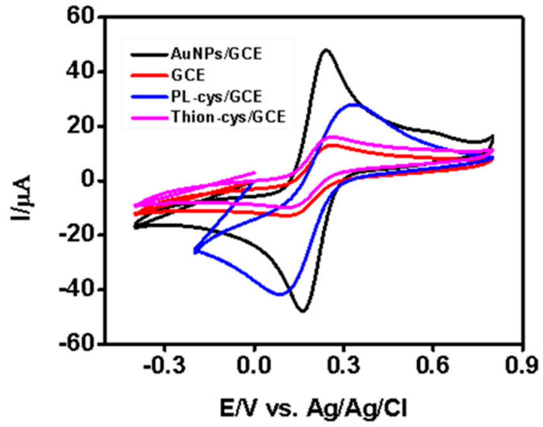

Figure S1. CV response of different modified electrodes in Fe^{3+} and AFM_1

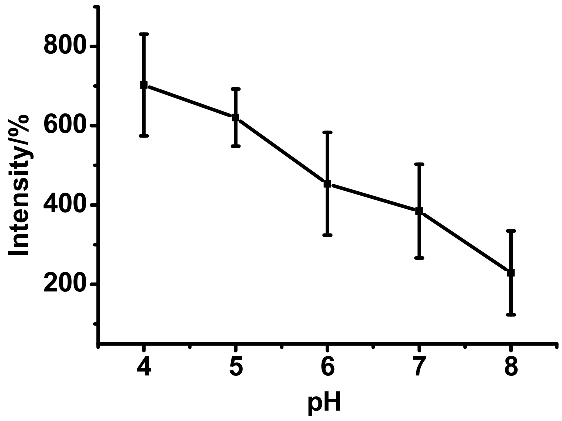

Figure S2. Effect of pH on fluorescence spectrum intensity.

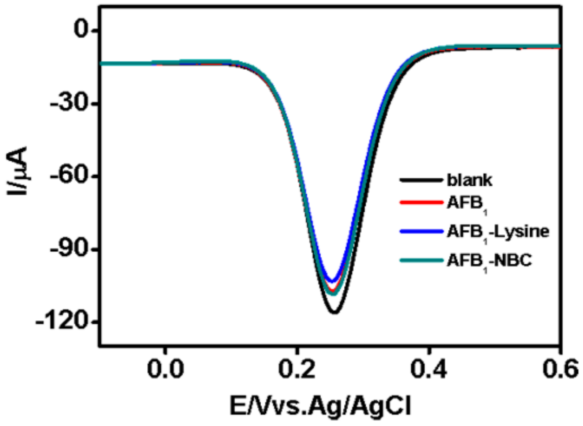

Figure S3. The DPV response of AFB1, AFB1-NAC, and AFB1-lysine respectively at AuNPs/GCE.

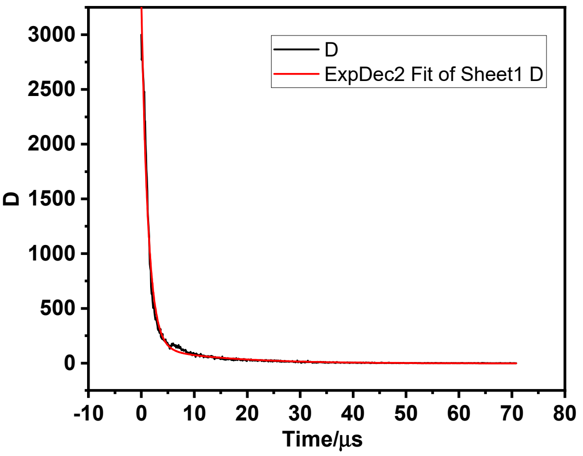

Figure S4. The second-order fitting curve of transient fluorescence spectra.

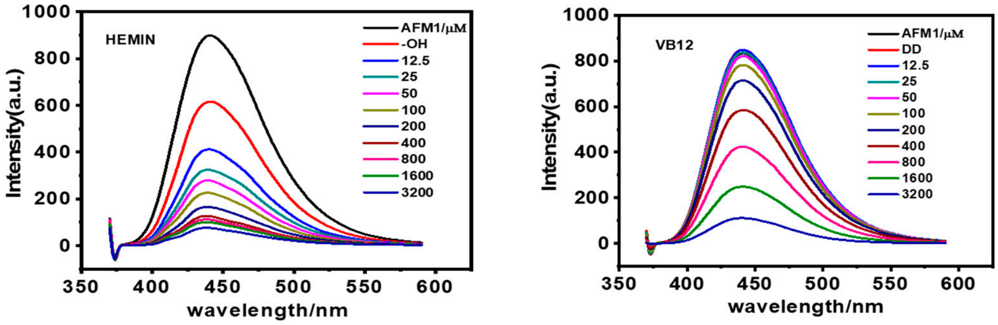

Figure S5. Fluorescence intensity at different concentrations of heme iron and VB_{12}.
